# Neurological Manifestations and their Correlated Factors in COVID-19 Patients; a Cross-Sectional Study

**DOI:** 10.22037/aaem.v9i1.1210

**Published:** 2021-04-27

**Authors:** Farzad Ashrafi, Davood Ommi, Alireza Zali, Sina Khani, Amirali Soheili, Mehran Arab-Ahmadi, Behdad Behnam, Shabnam Nohesara, Farbod Semnani, Alireza Fatemi, Mehri Salari, Reza Jalili khoshnood, Mohammad Vahidi, Niloofar Ayoobi-Yazdi, Saeed Hosseini Toudeshki, Elham Sobhrakhshankhah

**Affiliations:** 1 Functional Neurosurgery Research Center, Shohadaye Tajrish Neurosurgical Center of Excellence, Shahid Beheshti University of Medical Sciences, Tehran, Iran.; 2 Student Research Committee, School of Medicine, Shahid Beheshti University of Medical Sciences, Tehran, Iran.; 3 Advanced Diagnostic and Interventional Radiology Research Center, Tehran University of Medical Sciences, Tehran, Iran.; 4 Department of Internal Medicine, Iran University of Medical Sciences, Tehran, Iran.; 5 Mental Health Research Center, Iran University of Medical Sciences, Tehran, Iran.; 6 Students’ Scientific Research Center, Tehran University of Medical Sciences, Tehran, Iran.; 7 Department of Infectious Diseases, Shohadaye Tajrish Hospital, Shahid Beheshti University of Medical Sciences, Tehran, Iran.; 8 Department of Radiation Oncology, Iran University of Medical Sciences, Tehran, Iran.

**Keywords:** COVID-19, Neurologic Manifestations, Blood cell count, Risk Factors

## Abstract

**Introduction:**

COVID-19 might present with other seemingly unrelated manifestations; for instance, neurological symptoms. This study aimed to evaluate the neurologic manifestations and their correlated factors in COVID-19 patients.

**Methods:**

This retrospective observational study was conducted from March 17, 2020 to June 20, 2020 in a tertiary hospital in Iran. The study population consisted of adult patients with a positive result for COVID-19 real-time reverse transcriptase polymerase chain reaction (RT-PCR) using nasopharyngeal swabs. Both written and electronic data regarding baseline characteristic, laboratory findings, and neurological manifestations were evaluated and reported.

**Results:**

727 COVID-19 patients with the mean age of 49.94 ± 17.49 years were studied (56.9% male). At least one neurological symptom was observed in 403 (55.4%) cases. Headache (29.0%), and smell (22.3%) and taste (22.0%) impairment were the most prevalent neurological symptoms, while seizure (1.1%) and stroke (2.3%) were the least common ones. Patients with neurological manifestations were significantly older (p = 0.04), had greater body mass index (BMI) (p = 0.02), longer first symptom to admission duration (p < 0.001) and were more frequently opium users (p = 0.03) compared to COVID-19 patients without neurological symptoms. O_2 _saturation was significantly lower in patients with neurological manifestations (p = 0.04). In addition, medians of neutrophil count (p = 0.006), neutrophil-lymphocyte ratio (NLR) (p = 0.02) and c-reactive protein (CRP) (p = 0.001) were significantly higher and the median of lymphocyte count (p = 0.03) was significantly lower in patients with neurological manifestations.

**Conclusion:**

The prevalence of neurological manifestations in the studied cases was high (55.4%). This prevalence was significantly higher in older age, grated BMI, longer lasting disease, and opium usage.

## Introduction

Several studies showed clinical characteristics of the “coronavirus disease 2019 (COVID-19)” disease. In a systematic review by Leiwen Fu et al., fever (83.3%) cough (60.3%) and fatigue (38.0%) were the most common clinical symptoms (1). Respiratory tract infections and pneumonia have been commonly observed in infected patients. However, in some occasions, they show neurological alterations and signs including seizure, status epilepticus, impairment of consciousness, and encephalopathies (2). Besides, a study reported that some patients without typical symptoms of COVID-19 including fever, cough, anorexia, and diarrhea presented neurological manifestations as the initial symptoms such as dizziness, headache, impaired consciousness, acute cerebrovascular disease, ataxia, and seizure (3). These neurological symptoms were also seen in SARS-CoV infection, which caused polyneuropathy, encephalitis, and ischemic stroke and in MERS-CoV infection, which caused disturbance of consciousness, paralysis, ischemic stroke, Guillain-Barre syndrome, and seizure (4). Although there is no unanimity regarding the underlying mechanisms, it is probably due to exaggerated cytokine responses and/or the subsequent hypercoagulopathy in vessels, which cause anosmia, stroke, paralysis, cranial nerve deficits, encephalopathy, delirium, meningitis, and seizure (5). Due to lack of comprehensive and sufficient evidence concerning COVID-19 and its neurological manifestations, hereby, we report the characteristic neurological manifestations of SARS-CoV-2 infection in patients with a laboratory-confirmed diagnosis of COVID-19 and investigate the association between neurological symptoms and baseline characteristics as well as laboratory findings.

## Methods

***Study Design and Settings***

This retrospective observational study was conducted in Shohadaye Tajrish Hospital, a tertiary academic hospital located in Tehran, Iran. This hospital is among the major designated centers for COVID-19 patients during the pandemic. From March, 17, 2020 to June 20, 2020 all suspected individuals aged >18 years presenting with typical COVID-19 symptoms were tested for COVID-19 using a throat swab. Then, throat swab samples were put into 150 μL viral protective solution for further molecular examinations. Based on the WHO interim guidance (6), a confirmed case of COVID-19 is defined as a person with a positive result for RT-PCR test for SARS-CoV-2. The current study was performed according to the principles of the Declaration of Helsinki. An ethical approval was obtained from ethics committee of Shahid Beheshti University of Medical Sciences (Ethics code: IR.SBMU.RETECH.1399.115)

***Participants***

This study included all patients aged 18 years and above admitted to the hospital with typical COVID-19 symptoms and a positive throat swab COVID-19 test in evaluations. Convenience sampling method was used. Patients with previous neurocognitive disorders such as Alzheimer's disease and patients who were unconscious at the time of admission were excluded from the study. 

**Data gathering**

Demographic data (age, sex, body mass index (BMI)), history of comorbid diseases (diabetes mellitus, hypertension, chronic kidney disease, cardio and cerebrovascular disease, cancer), clinical symptoms (fever, dyspnea, cough, gastrointestinal discomforts, sore throat), laboratory findings, and neurological manifestations were gathered for all patients using hospital’s both electronic and written records. If there was missing data, data clarification was performed via phone call or consulting with related physicians. Neurological manifestations were categorized into three classes: I. Symptoms related to skeletal muscular injury; II. Central nervous system (CNS) manifestations including headache, drowsiness, convulsion, ataxia, impaired consciousness, and acute cerebrovascular disease; III. Peripheral nervous system (PNS) features such as nerve root pains and cranial nerve symptoms such as anosmia, ageusia, visual discomforts, and photophobia. 

Critical cases were defined as those admitted to intensive care unit (ICU) with acute respiratory distress syndrome (ARDS) criteria consisting of severe dyspnea, respiratory rate ≥30/minute, blood oxygen saturation ≤80%, PaO2/FiO2 ratio and unstable vital signs (7). Trained medical students were responsible for data gathering.

**Statistical analysis**

Data were presented as mean ± standard deviation (SD) for normally distributed, median (Q1 – Q3) for skewed variables, and frequency (percent) for categorical data. Normality assumption was checked using Kolmogorov-Simonov test. Mean and median differences were tested using independent T-test and Mann-Whitney U test, respectively. The distribution of categorical data was assessed using chi-square test (with exact p-value). P<0.05 was considered as significance level and all statistical analyses were performed using IBM SPSS Statistics 23.

## Results

**Figure 1:**
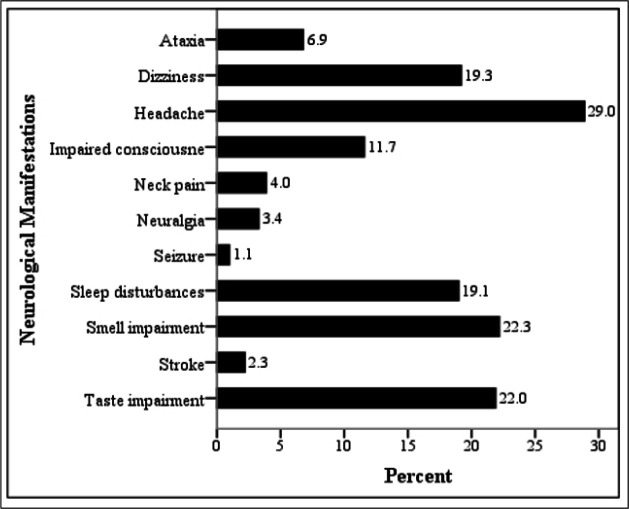
The frequency of neurological manifestations in patients with COVID-19

727 COVID-19 patients with the mean age of 49.94 ± 17.49 years (22 - 91) were enrolled (56.9% male). At least one neurologic symptom was observed in 403 (55.4%) cases. Figure 1 displays the prevalence of neurological manifestations in the studied participants. Headache (29.0%), and smell (22.3%) and taste impairment (22.0%) were the most prevalent neurologic symptoms, while seizure (1.1%) and stroke (2.3%) were the least common ones. 

Table 1 compares the baseline characteristics and laboratory findings of studied cases between cases with and without neurological manifestations. Patients with neurological manifestations were significantly older (p = 0.04), had greater BMI (p = 0.02), longer first symptom to admission duration (p < 0.001) and were more frequently opium users (p = 0.03) compared to COVID-19 patients without neurological symptom. O_2 _saturation was significantly lower in patients with neurological manifestations (p = 0.04). In addition, medians of neutrophil count (p = 0.006), neutrophil-lymphocyte ratio (NLR) (p = 0.02) and c-reactive protein (CRP) (p = 0.001) were significantly higher and the median of lymphocyte count (p = 0.03) was significantly lower in patients with neurological manifestations.

85 (11.7%) cases were critically ill and admitted to ICU. Table 2 compares the prevalence of neurologic manifestations between stable and critically ill patients. The prevalence of neurologic manifestations was not significantly different between stable and critically ill patients except for headache and dizziness, which were higher in stable patients (30.2% vs 20.0%, p=0.049 and 20.6% vs 9.4%, p= 0.014, respectively).

## Discussion

Herein, neurological symptoms of 727 hospitalized patients with COVID-19 were evaluated. Overall, 403 (55.4%) patients had at least one of the reported neurological symptoms. Among all neurological symptoms, headache (29.0%) and smell impairment (22.3%) were the most prevalent ones. Neutrophil count, NLR, and CRP were significantly higher in patients with neurological manifestations. Moreover, the prevalence of neurologic manifestations was not significantly different between stable and critically ill patients except for headache and dizziness, which were more prevalent in stable patients.

According to the literature, among gastrointestinal disturbances in COVID-19, anorexia is the most prevalent one (8), which is parallel to our findings. Anorexia is probably due to the release of inflammatory cytokines along with the adverse effects of various drugs consumed by these patients. Headache is also a prominent feature in COVID-19 patients, which has been described among the most prevalent neurological symptoms by many studies (3, 9-11). It may occur due to systemic inflammation or virus invasion to brain blood vessels. About a quarter of patients reported smell and taste impairment without significant difference between critical and stable groups. We found that patients with neurological symptoms were significantly older (p = 0.005), had higher BMI (p = 0.02), longer hospitalization (p = 0.002), and longer first symptom to admission duration (p < 0.001) compared to COVID-19 patients without neurological symptoms. 

In a study in China, it was shown that obese patients were at greater risk of developing severe COVID-19 infection compared to normal-weight patients and this is of great importance, because it may indeed lead to increased hospitalization and worse clinical outcomes (12). 

The prevalence of neurologic manifestations was not significantly different between stable and critically ill patients except for headache and dizziness, which were more prevalent in stable patients. This may be related to the fact that critically ill patients are commonly bedridden and headache and dizziness are commonly triggered by walking. 

Up to now, several studies have been performed to identify and characterize COVID-19 pathophysiologic mechanisms leading to its neurological manifestations. A study by M. Fotuhi et al. categorized neurological symptoms into a conceptual framework of their own so-called “NeuroCovid Staging”, which included 3 stages. In the proposed NeuroCovid Stage I infection with SARS-CoV2 is limited to nasal and gustatory epithelial cells and the cytokine storm is limited. Then, in the NeuroCovid Stage II, there is a vigorous cytokine storm resulting in a hypercoagulable state, which is also responsible for blood clot formation and higher probability of strokes in these patients. Finally, during the NeuroCovid Stage III, virus-induced cytokine storm damages the blood brain barrier resulting in the penetration of inflammatory factors and consequent severe complications including delirium, encephalopathy, and seizure (5). In a recent review by Ellul et al., it was stated that acute ischemic stroke might occur due to a destabilized carotid plaque or as a result of atrial fibrillation. Viral replication in the cerebral blood vessels could be a possible reason for such manifestations (13). 

It seems that COVID-19 is also related with chronic neurologic complications, particularly a greater risk of stroke, even in youths. In a case-series by Ashrafi et al. all six COVID-19 patients presenting with stroke were younger than 55 and did not have any major risk factors for stroke (14). 

Moreover, COVID-19 patients are more prone to anxiety, which is even more prevalent among patients with preexisting comorbidities such as Parkinson. Further studies should be performed to evaluate the correlation between neurological comorbidities and anxiety during the COVID-19 pandemic (15).

**Table 1:** Comparing the baseline characteristics and laboratory findings of COVID-19 patients between cases with and without neurological manifestations

**Variables**	**Neurological manifestation**		**p**
	**No (n = 324)**	**Yes (n = 403)**	
**Age (year)**			
Mean ± SD	48.50 ± 17.31	51.10 ± 17.57	0.04
**Sex**			
Female	138 (42.6)	175 (43.4)	0.82
Male	186 (57.4)	228 (56.6)	
**BMI (kg/m** ^2^ **)**			
Mean ± SD	25.71 ± 4.52	26.54 ± 4.26	0.02
**Habit**			
Smoke (yes)	42 (13.0)	39 (9.7)	0.16
Ex-Smoke (yes)	20 (6.2)	38 (9.4)	0.11
Alcohol (yes)	8 (2.5)	6 (1.5)	0.34
Opium (yes)	2 (0.6)	11 (2.7)	0.03
**Hospitalization (day)**	1 (0 – 4)	1 (0 – 4)	0.22
**First Symptom to Admission (day)**	2 (1 – 4)	4 (2 – 8)	<0.001
**Vital signs**			
Heart Rate (/minute)	87.92 ± 13.71	88.56 ± 14.48	0.15
RR (/minute)	18.02 ± 3.53	18.72 ± 7.12	0.09
Temperature (Celsius)	37.32 ± 0.77	37.30 ± 0.79	0.70
O2 saturation (%)	94 (91 – 96)	93 (90 – 96)	0.04
SBP (mmHg)	116.68 ± 13.00	118.51 ± 14.35	0.08
DBP (mmHg)	74.27 ± 9.26	74.61 ± 9.33	0.64
**Laboratory findings**			
WBC (/µL)	6.2 (4.4 – 8.5)	5.8 (4.6 – 8.1)	0.96
Hemoglobin (g/dL)	13 (11.8 – 14)	13 (11.8 – 14.3)	0.55
Platelet (/µL)	179 (139 – 214)	166 (131– 223)	0.54
Neutrophil (/µL)	7.0 (6.0 – 8.0)	7.5 (6.5 – 8.3)	0.006
Lymphocyte (/ µL)	2.5 (1.5 – 3.3)	2.1 (1.5 – 3.0)	0.03
NLR	2.8 (1.9 – 5.3)	3.5 (2.2 – 5.7)	0.02
PLR	1.3 (0.9 – 1.7)	1.3 (0.9 – 1.9)	0.12
Cr (mg/dL)	1.0 (0.9 – 1.3)	1.1 (1.0 – 1.4)	0.06
BUN (mg/dL)	13 (11– 17.8)	14 (11 – 18)	0.31
CRP (mg/dL)	15.0 (7.4– 34.0)	25.0 (11 – 45.3)	0.001
CPK (IU/L)	110 (60.3 – 162.8)	118 (61 – 256)	0.13
LDH (U/L)	495 (373 – 599)	495 (399 – 629)	0.55

Data are presented as mean ± standard deviation, median (Q1 – Q3), or frequency (%). BMI: body mass index; RR: respiratory rate; SBP: systolic blood pressure; DBP: diastolic blood pressure; WBC: white blood cells; NLR: neutrophil-to-lymphocyte ratio; PLR: platelet-to-lymphocyte ratio; Cr: creatinine; BUN: blood urea nitrogen; CRP: c-reactive protein; CPK: creatine phosphokinase; LDH: lactate dehydrogenase.

**Table 2:** Comparison of neurological manifestations of COVID-19 patients between critically ill patients and others

**Variables**	**Critically ill**		**P**
	**No (n = 642)**	**Yes (n = 85)**	
Neck‎ pain	25 (3.9)	4 (4.7)	0.77
Headache	194 (30.2)	17 (20.0)	0.05
Impaired consciousness	72 (11.2)	13 (15.3)	0.27
Dizziness	132 (20.6)	8 (9.4)	0.014
Ataxia	48 (7.5)	2 (2.4)	0.08
Seizure	6 (0.9)	2 (2.4)	0.24
Smell impairment	147 (22.9)	15 (17.6)	0.27
Taste impairment	143 (22.3)	17 (20.0)	0.63
Sleep disturbances	124 (19.3)	15 (17.6)	0.82
Neuralgia	21 (3.3)	4 (4.7)	0.52
Stroke	13 (2.0)	4 (4.7)	0.13
Total*	362 (56.4)	41 (48.2)	0.16

*At least one of the measured neurological symptoms was observed. Data are presented as frequency (%).

This might be the result of SARS-CoV-2 directly attacking the CNS as the virus was also found in the CSF fluid. Cytokine storm may also be responsible for these neurological symptoms particularly by resulting in a hypercoagulable state. 

## Conclusion:

According to our findings, more than half of COVID-19 patients had at least one of the studied neurological symptoms. This prevalence was significantly higher in older age, grated BMI, longer lasting disease, and opium usage.

## Limitations

Some of the neurological features such as encephalitis, encephalopathy, acute disseminated encephalomyelitis (ADEM) and myelitis might not be diagnosed at the time of hospitalization as they will manifest later. However, in cases with impaired consciousness, infection with SARS-CoV-2 is not the only trigger and other possible causes like drugs, toxins and metabolic derangement should also be considered. Furthermore, admission to the intensive care unit is an important risk factor, as critical states like sepsis may result in neurological manifestations such as encephalopathy.
